# *SHROOM3*, the gene associated with chronic kidney disease, affects the podocyte structure

**DOI:** 10.1038/s41598-020-77952-9

**Published:** 2020-12-03

**Authors:** Ryo Matsuura, Atsuko Hiraishi, Lawrence B. Holzman, Hiroki Hanayama, Koji Harano, Eiichi Nakamura, Yoshifumi Hamasaki, Kent Doi, Masaomi Nangaku, Eisei Noiri

**Affiliations:** 1 Department of Nephrology and Endocrinology, The University of Tokyo Hospital, 7-3-1 Hongo, Bunkyo-ku, Tokyo, 113-8655 Japan; 2 Division of Genomic Medicine and Disease Prevention, Institute of Medical Science, The University of Tokyo, Shirokanedai, 4-6-1 Minato-ku, Tokyo, 108-8639 Japan; 3 Renal Electrolyte and Hypertension Division, Perelman School of Medicine, University of Pennsylvania, Philadelphia, 19104 USA; 4 Department of Chemistry, The University of Tokyo, 7-3-1 Hongo, Bunkyo-ku, Tokyo, 113-0033 Japan; 5 Department of Hemodialysis and Apheresis, The University of Tokyo Hospital, 7-3-1 Hongo, Bunkyo-ku, Tokyo, 113-8655 Japan; 6 Department of Acute Medicine, The University of Tokyo Hospital, 7-3-1 Hongo, Bunkyo-ku, Tokyo, 113-8655 Japan; 7 National Center Biobank Network, National Center for Global Health and Medicine, 1-21-1 Toyama, Shinjuku, Tokyo, 162-8655 Japan

**Keywords:** Podocytes, RNA

## Abstract

Chronic kidney disease is a public health burden and it remains unknown which genetic loci are associated with kidney function in the Japanese population, our genome-wide association study using the Biobank Japan dataset (excluding secondary kidney diseases, such as diabetes mellitus) clearly revealed that almost half of the top 50 single nucleotide polymorphisms associated with estimated glomerular filtration rate are located in the *SHROOM3* gene, suggesting that *SHROOM3* will be responsible for kidney function. Thus, to confirm this finding, supportive functional analyses were performed on *Shroom3* in mice using fullerene-based siRNA delivery, which demonstrated that *Shroom3* knockdown led to albuminuria and podocyte foot process effacement. The in vitro experiment shows that knockdown of *Shroom3* caused defective formation of lamellipodia in podocyte, which would lead to the disruption of slit diaphragm. These results from the GWAS, in vivo and in vitro experiment were consistent with recent studies reporting that albuminuria leads to impairment of kidney function.

## Introduction

Chronic kidney disease (CKD) is a public health burden with high prevalence of more than 10% and increasing incidence^1^. Defined based on a decrease in estimated glomerular filtration rate (eGFR) to less than 60 mL/min/1.73 m^2^ as sustained abnormality of kidney dysfunction^2^, CKD can progress to end-stage renal disease (ESRD), and increase cardiovascular risk and mortality^3–6^. A Japanese observational study in Okinawa demonstrated that higher proteinuria lead to faster renal deterioration towards ESRD^7^. However, renal functional deterioration needs to be accounted for in greater detail through functional analysis of genetic factors involved.

It has been assumed that the variation in kidney function level is associated with genetic factors^8^. Recently, genome-wide association studies (GWAS) identified several genetic loci associated with indices of kidney function^9,10^. However, most of these studies included diabetes mellitus in their analyses, given its enormous impact. Therefore, this study attempted to exclude diabetes to find more primary factors responsible for kidney function decline. In addition, given that earlier analyses were conducted in Western populations, it remains unknown which genetic loci are associated with kidney function in the Japanese population. In the present study, we conducted GWAS in a Japanese cohort with the quantitative trait loci (QTL) analysis using the estimated glomerular filtration rate (eGFR) as the continuous dependent variable. Our GWAS analysis of eGFR QTL identified a greater number of tag variants related to shroom family member 3 (*SHROOM3*) in this cohort than that reported in previous studies^8,9,11^. Therefore, we conducted functional analysis on the *Shroom3* gene to examine its potential pathological role using fullerene-based siRNA in vivo delivery, tetra(piperazino)fullerene epoxide (TPFE)^12^.

## Results

### Genetic loci associated with eGFR in humans

**Figure 1:**
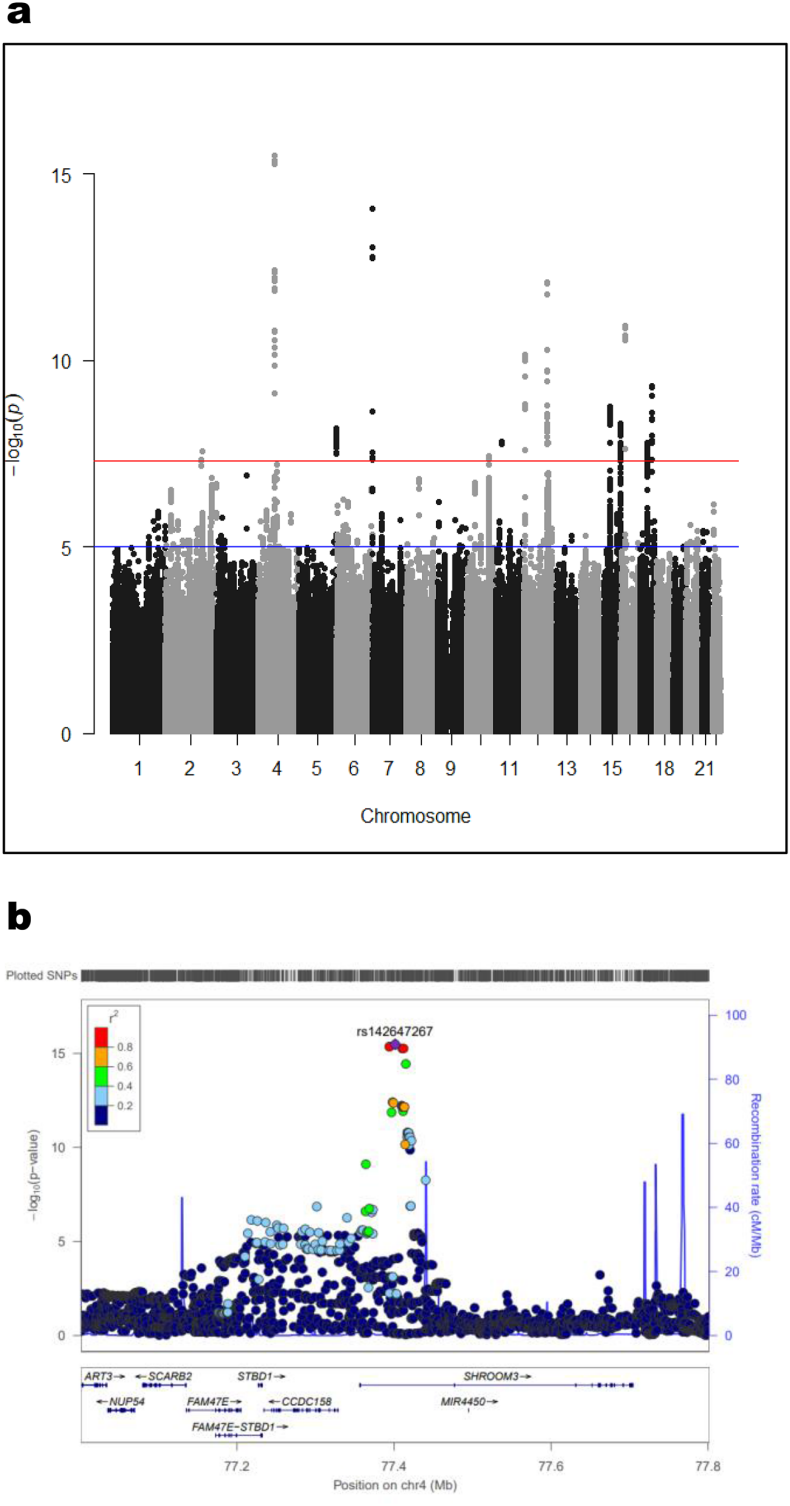
GWAS results. (**a**) Overviews of the GWAS for eGFR. Shown are the − log_10_ (*P* values) of the SNPs for eGFR. The genetic loci that satisfied the genome-wide significance threshold of *P* < 5.0 × 10^−8^ (red horizontal line) above the GWAS. (**b**) The genome-wide significant susceptibility loci in *SHROOM3* region for eGFR. − log10 *P* values are plotted versus genomic position. The most significant SNP in each region is plotted in purple. Linkage disequilibrium (LD) based on the HapMap CEU sample is color-coded red (r2 to top SNP 0.8–1.0), orange (0.6–0.8), green (0.4–0.6), light blue (0.2–0.4) and deep blue (< 0.2).

This study population included 15.2% of patients with CKD defined as eGFR < 60 mL/min/1.73 m^2^. Figure 1a summarizes the results for eGFR and top 50 SNPs associated with eGFR in eQTL analysis (see also Supplemental Table 1). Of the 50 SNPs identified, 21 are located in the *SHROOM3* gene, with rs142647267 shown to be the most significant (Fig. 1b). Thus, assuming that *SHROOM3* had a major role in maintaining normal kidney function, we went on to investigate the function of *Shroom3*.

### Determination of a reagent-to-base pair ratio for gene knockdown in podocytes

We evaluated the function of *Shroom3* using siRNA and its in vivo delivery, TPFE. First, to investigate the proper ratio of TPFE to siRNA, we conducted a control experiment to obtain optimal conditions for knockdown *Gpc5* in podocyte, the gene previously proved to be associated with nephrotic syndrome and localized in podocytes^13,14^. When si*Gpc5*-TPFE complexes was injected at a base-pair ratio (R) of 10, no staining was shown in areas previously identified for *Gpc5* staining with anti-*Nephrin* antibody in podocytes (Supplemental Figure 1). On the other hand, podocytes showed weak staining for *Gpc5* when si*Gpc5*-TPFE complexes was injected at an R of 15, suggesting that siRNA and TPFE is best mixed at an R of 10 to knockdown the genes in podocytes.

### tdTomato-Podocin receptor mice

These mice were generated by the breeding of Podocin-Cre and *Gt(ROSA)26Sortm9(CAG-tdTomato)Hze* mice. As a promoter fragment of the human *NPH2* gene was reported to direct podocyte-specific transgene expression in mice, the same approach was used here with the Cre-Lox strategy, which led to the red signal of tdTomato being predominantly expressed in podocytes (Supplemental Figure 2).

**Figure 2:**
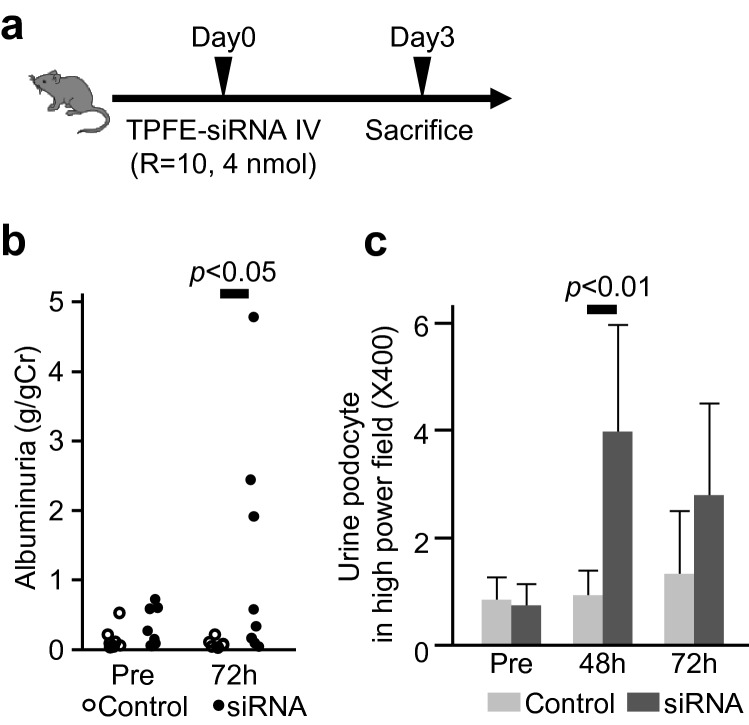
The association with si-*Shroom3* injection and albuminuria. (**a**) The scheme of the experiment using tdTomato-Podocin reporter mice. TPFE-siRNA complexes were injected and sacrificed three days later. (**b**) Albuminuria before and after TPFE-siRNA complexes injection. Before injection, urinary albumin was not different between mice with scramble siRNA and si-*Shroom3*. Urinary albumin significantly increased in 72 h post si-*Shroom3* injection while urinary albumin did not increase in mice with scramble siRNA. (**c**) Urinary podocytes per high field were more in mice with si-*Shroom3* than in mice with negative control siRNA. n = 5 for each.

### Shroom3 knockdown caused albuminuria through foot process effacement

**Figure 3:**
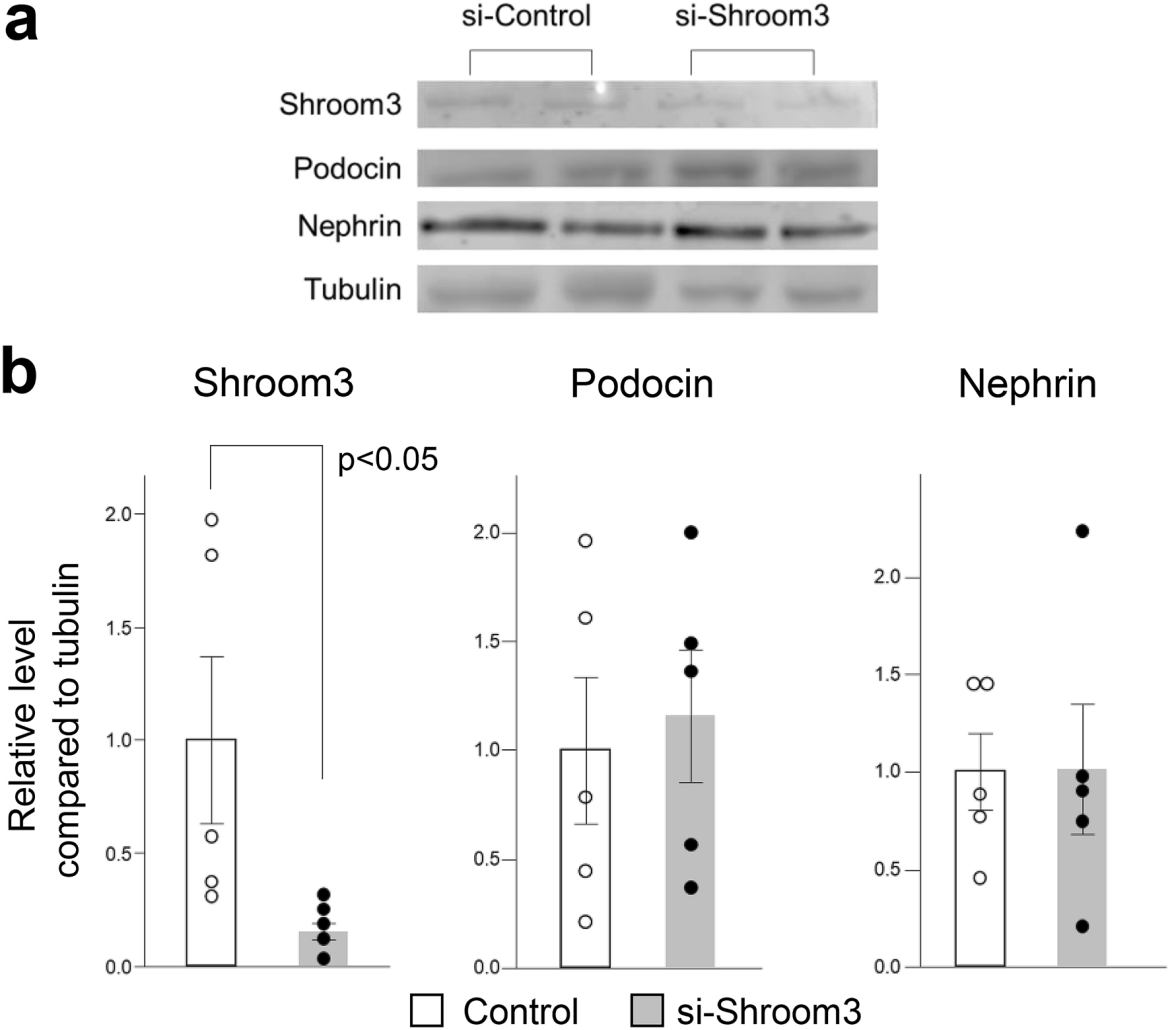
The protein level of *Shroom3,* Podocin and Nephrin in kidney. (**a**) The representative image of Western blotting showing protein level of Shroom3 (top), Podocin (upper middle), Nephrin (lower middle) and Tubulin (bottom). (**b**) Quantitative evaluation of each protein level in the scramble siRNA and si-Shroom3 groups. Protein level of Shroom3 was reduced in si-Shroom3 treated group while podocin and nephrin were unchanged. n = 5 for each.

**Figure 4:**
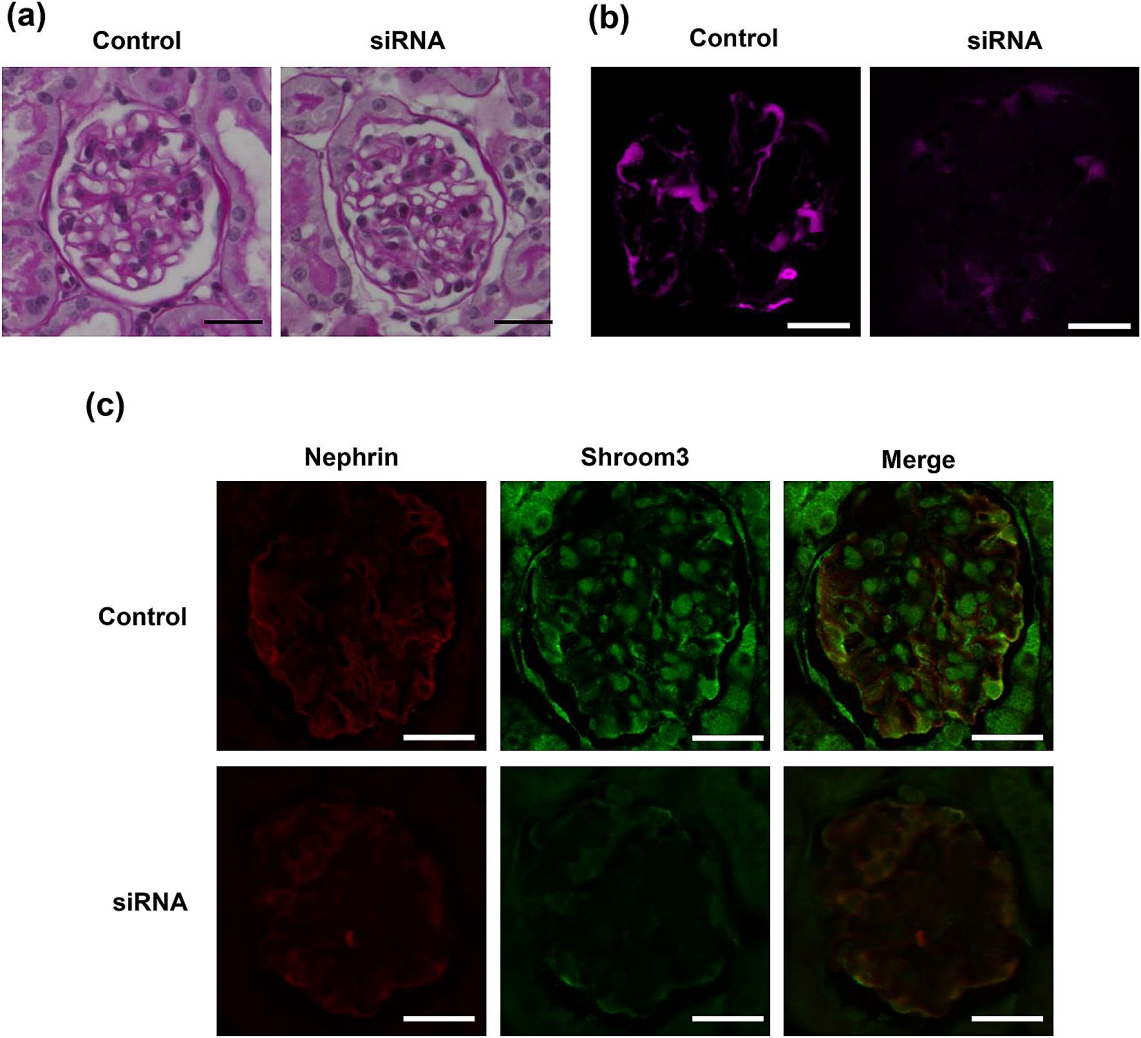
The pathology of kidney in mice with scramble siRNA and si-*Shroom3*. (**a**) No apparent changes in periodic acid-Schiff (PAS) staining were seen in glomeruli of mice with scramble siRNA and si-*Shroom3*. (**b**) Podocin detected as tdTomato (pink) decreased in si-*Shroom3* mice. (**c**) Immunofluorescence staining revealed that the area stained by nephrin (red) and *Shroom3* (green) were decreased in si-*Shroom3* knockdown. Bar in all figures denotes 25 µm.

To investigate the role of *Shroom3* further, functional experiments were conducted to investigate if the reporter gene predominantly expressed in podocytes can be abrogated with the knockdown of *Shroom3*. To this end, the tdTomato-Podocin reporter mice were injected with TPFE-siRNA complexes containing 4 nmol si-*Shroom3* or control siRNA and were sacrificed after 72 h (Fig. 2a). Albuminuria appeared 72 h after injection of si-*Shroom3*-TPFE complexes (Fig. 2b). Podocytes were more excreted in urine in the si-*Shroom3* group than in the control group (Fig. 2c). *Shroom3* mRNA expression in the whole kidney was reduced, though not significant level, in the si-*Shroom3* group, while no differences were noted in the expression of *Shroom3* mRNA in other tissues between in the si-*Shroom3* and the control groups (Supplemental Figure 3). Immunoblot also showed that protein expression of Shroom3 was reduced in kidneys of the si-*Shroom3* group while protein expressions of nephrin and podocin were unchanged (Fig. 3a,b). Again, while no changes were noted in glomerular appearance of both groups on light microscopy (Fig. 4a), tdTomato-Podocin signals were shown to be fewer in the podocytes of the mice receiving si-*Shroom3*-TPFE complexes but were clearly detected in those of the control mice (Fig. 4b). Colocalized immunofluorescence staining of *Shroom3* and Nephrin was seen in the podocytes of control mice but not in those of mice injected with si-*Shroom3*-TPFE complexes (Fig. 4c).

**Figure 5:**
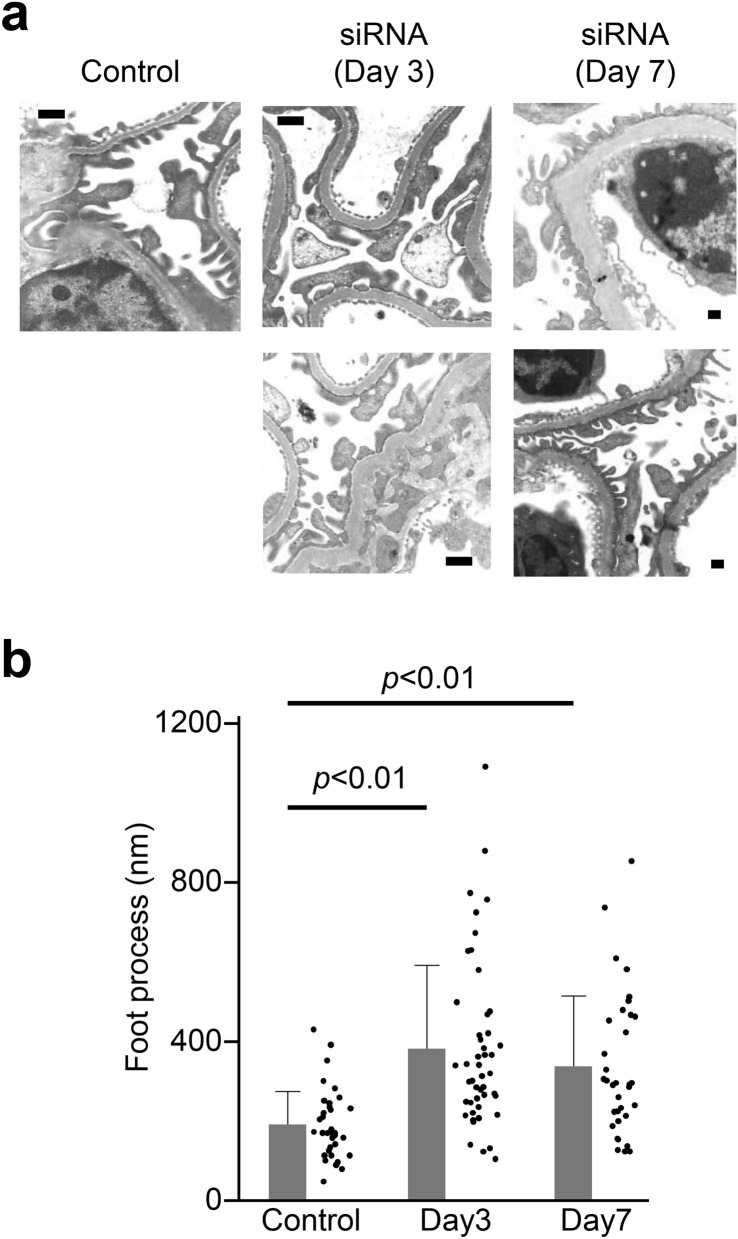
Foot process effacement in electron microscopy. (**a**) Electron microscopy conducted on day 3 revealed virtually foot-process effacement in mice with si-*Shroom3*. (**b**) Quantitative evaluation of foot process length confirmed the foot process effacement in mice with si-*Shroom3*. Forty podocyte process in each group were measured.

On transmission electronic microscopy, foot process effacement was seen in the mice injected with si-*Shroom3*-TPFE complexes on days 3 and 7, while the foot processes remained intact in the mice injected with si-control-TPFE complexes (Fig. 5).

### Shroom3 knockdown led to the failure of podocyte lamellipodia formation

**Figure 6:**
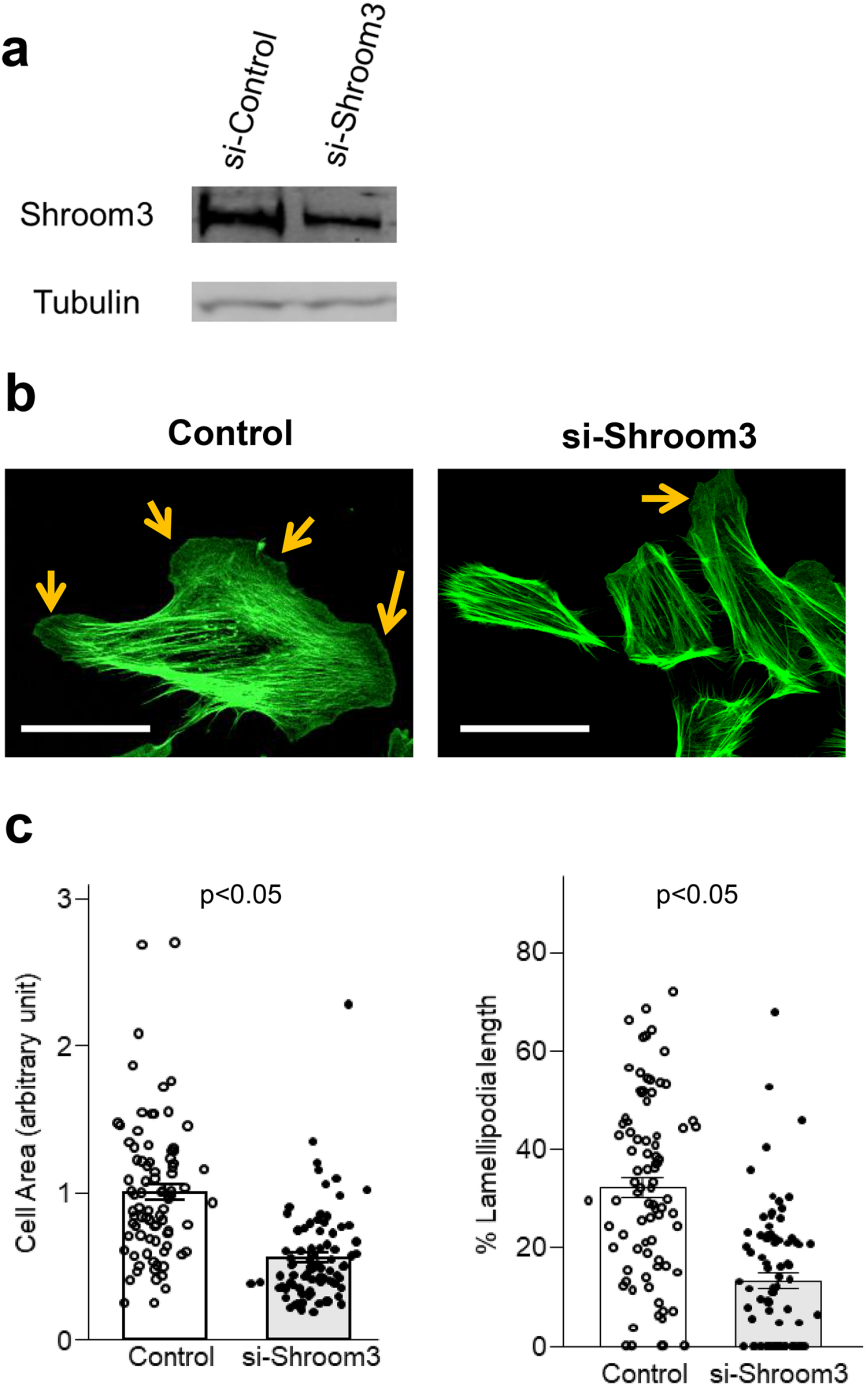
Shroom3 knockdown decreased podocyte lamellipodia. Rat glomerular epithelial cells transfected with either Stealth RNAi Negative Control Med GC (Invitrogen, Carlsbad, CA) or with predesigned Shroom3 siRNA were used. Western blotting and immunofluorescence were performed at 48 h after knockdown of Shroom3. (**a**) By Western blotting, the reduced level of Shroom3 was confirmed in si-Shroom3 treated cells. (**b**) Representative images of cell shape and phalloidin in scramble siRNA- and si-Shroom3-treated cells. Scramble siRNA-treated cells are larger and lamellipodia were well formed while si-Shroom3-treated cells are small and less lamellipodia were formed. (**c**) Quantitative analysis of cell area and lamellipodia formation. The result is consistent with (**b**) representative images.

We did the in vitro experiment to evaluate how Shroom3 knockdown affect podocyte structure. At 48 h after Shroom3 knockdown on rat glomerular epithelial cells using Lipofectamine technique, confirming reduced protein expression of Shroom3 (Fig. 6a), we found that podocyte size became smaller and lamellipodia formation was defective in si-Shroom3 group (Fig. 6b,c).

## Discussion

This study evaluated the genetic risk of *SHROOM3* for non-diabetic CKD with GWAS and functional analysis of *Shroom3* in podocytes in vivo with new siRNA delivery technology. We found in this study that (1) *SHROOM3* was among the genes remarkably associated with non-diabetic CKD in the Japanese population; (2) *Shroom3* has a role in podocyte structure development and its knockdown causes foot process effacement at podocytes and albuminuria; and (3) TPFE represents a new pharmaceutical carrier in nephrotic syndrome with its ability to deliver siRNA to podocytes.

Our GWAS study provided evidence for the genetic association of *SHROOM3* to the eGFR decliner. While a similar analysis has been conducted in a Japanese population of 14,539 as a discovery cohort, the role of *SHROOM3* has not been confirmed^15^. This is presumably due to differences in sample size and cohort. Furthermore, this study used the BBJ cohort of 46 diseases from which diabetes mellitus, cancer, thyroid diseases (e.g., Basedow disease), liver diseases, liver cirrhosis, hepatitis B, hepatitis C, cardiac insufficiency, and epilepsy had been excluded, given that these diseases and conditions could account for secondary kidney functional decline. Of these, diabetes mellitus is assumed to be a potential to hide the unique property to cause and effect link to eGFR decline. In this study, *SHROOM3* was shown to be present in 21 of the top 50 genes. Of these, those notably associated with CKD, such as MYL2, PDILT, and UNCX were shown to be preserved, thus providing support for the robustness of our analysis with RSQR values (imputation quality, over 0.9). The appearance in *SHROOM3* of tag SNPs of genome-wide significance was remarkably increased with this solution. The functional significance of top variants would be interested though 20 of 50 top genetic variant seen in eGFR decliners are dominated by *SHROOM3*. For instance, the top variant, rs142647267, locates at intron where the position often causes splicing variant (see Supplemental Figure 4). In vitro work of genome editing to prove each contribution or combination to functional difference may clarify more detail relationship to eGFR decline and further personalize the medical approach. Now, given that current results from larger Japanese cohorts are shown to be consistent with those from Western cohorts^9,10^, *SHROOM3* can be a unique gene associated with kidney function decline, regardless of races. Further studies are required to investigate whether mutant *SHROOM3* patients with renal impairment may be more likely to progress to CKD or whether a particular care bundle aimed at prevention of development of CKD may be effective in patients genetically at risk.

Of note, a previous animal experiment also showed that *Shroom3* knockout is associated with abnormal glomerulogenesis and shortening/thickening of the collecting duct in the process of kidney development^16^. Our experiment also found that *Shroom3* knockdown in adult mice led to foot process effacement without reducing the number of podocytes. The in vitro experiment also showed that *Shroom3* knockdown caused defective formation of lamellipodia in podocyte. Previous studies show that lamellipodia in podocyte is critical for structural integrity because it provides a lipid raft-membrane and recruits proteins necessary for slit diaphragm^36,37^. Therefore, *Shroom3* has a crucial role in podocyte structural integrity. Again, current results from our GWAS and in vivo experiment have led us to the hypothesis that albuminuria caused by mutant *Shroom3* leads to impairment of kidney function. Numerous experimental studies suggest that albumin leakage from glomeruli triggers a destructive effect and inflammatory response, leading to severe tubulointerstitial damage^17–20^. In addition, clinical studies confirm that albuminuria is associated with increased risk of ESRD^7,21,22^, suggesting that our study results are consistent with those from recent clinical and basic studies.

The deletion of a critical gene to podocytes is shown to result in an embryonic lethal phenotype that precludes functional analysis in mature podocytes. This is also the case with *Shroom3* and *Nephs1*. Eremina et al.^23^ generated mice expressing Cre recombinase in podocyte using 4.125-kb *Nephs1* promoter. However, they found that Cre recombinase was expressed in the brain in addition to podocytes. In contrast, the human *NPHS2* gene was truly driven in a podocyte-specific fashion^24^. Using NPHS2 as a promoter of Cre recombinase, this study successfully guided *Gt(ROSA)26Sortm9(CAG-tdTomato)Hze* to podocytes alone.

The main strengths of our study are not only that it revealed the role of *Shroom3* in podocyte structure but that it showed the utility of TPFE for siRNA delivery. Although RNA interference is expected as a therapeutic method, one of the challenges that remain to be overcome is to deliver short-interfering RNA (siRNA) to a target site with minimal off-target effects. Various molecular delivery platforms are currently being investigated to overcome this barrier. siRNA delivery platforms are expected to be biocompatible, nonimmunogenic, and allows siRNA to be delivered to any specific site^25–28^. However, while several studies reported candidate reagents for delivery of siRNA to the mesangium and tubules of the kidney^29,30^, none was shown to be capable of targeting podocytes. For example, in our study, Shroom3 is generally expressed in various organs such as brain, lung, heart and liver as well as kidney according to the published datasets on RNA or protein expression. Because Shroom3 has a crucial role in actin cytoskeleton, it is required for the platform to avoid delivering siRNA to other organ than kidney with effective knockdown in podocytes. In this regard, our study demonstrated that fullerene-siRNA complexes could be selectively delivered to podocytes using an organ specific base-pair ratio (discussed below), thus suggesting the potential of selective gene-silencing therapy. Moreover, TPFE is shown to have much lower *in vitro*^31,32^ and in vivo toxicity than the cationic lipid equivalents^31^. Thus, fullerene is expected to be an ideal siRNA delivery agent.

We have previously found that fullerene could target the lung alone with R = 20^33^. In this study, fullerene was shown to successfully target podocytes with R = 10. These results indicate that fullerene is capable of delivering siRNA to any specific target without off-target consequences when the fullerene-siRNA complex is adjusted to an appropriate ratio of the reagent to base-pair. No other additive to combine with fullerene for organ targeting was needed and thereby, creating TPFE-based drugs would involve no appreciable cost consideration. Furthermore, it would readily allow for organ-specific RNA knockdown in adult animals and the generation of knockout animals with several disadvantages to be effectively obviated.

In summary, we showed the genetic association of *SHROOM3* with progressing kidney dysfunction, elucidated that *Shroom3* has a role in maintaining podocyte integrity thus avoiding albumin leakage and that siRNA-TPFE complexes have the potential tool to knockdown podocyte-specific genes in animal experiments for the proof of concept in podocyte-related pathophysiological conditions.

## Methods

### GWAS

For the discovery GWAS analysis, we used the BioBank Japan (BBJ) cohort^34^. BBJ is a registry of patients diagnosed with 47 common diseases. Of the 182,000 participants with genome-wide SNP typing data, we excluded patients with diabetes mellitus, cancer, thyroid diseases (e.g., Basedow disease), liver diseases, liver cirrhosis, hepatitis B, hepatitis C, cardiac insufficiency, and epilepsy, because of their potential interference with creatinine levels. In addition, those less than 18 and over 70 years were excluded. Thus a total of 41,830 subjects (22,158 males, 19,672 females) were available for current analyses. The principal components analysis (PCA) was conducted using Plink 1.9, and first 4 PC eigenvectors were used as covariates in the analyses. For all missing genotypes, we used 1000 Genomes project cosmopolitan reference panel (phase 1), and the data was generated using MaCH 1.0.18 software. Association analysis with eGFR as a continuous variable under an additive model was performed using linear regression with adjustment for age, sex, and 4 principal components as described above. We used Plink1.9 for genotyped SNPs and ProbABEL (ver 0.5.0) for imputed data for the statistical analyses. Variants with a minor allele frequency (MAF) of more than (or equal to) 0.05 were considered for analysis. The Manhattan plots were drawn with the “qqman” function in R. Regional plots of association were created with LocusZoom (https://genome.sph.umich.edu/wiki/LocusZoom_Standalone). The genome-wide significance levels were set at *P* < 5 × 10^–8^, and suggestive levels at *P* < 5 × 10^–6^ in the analysis. Lead SNPs were defined as those reaching the smallest *P* value in each genetic locus defined as the position on a chromosome.

### Mice

All animal experiments were conducted in accordance with the NIH Guide for the Care and Use of Laboratory Animals (U.S. Department of Health and Human Services, Public Health Services, National Institute of Health, NIH publication no. 86–23, 1985), and approved by the Ethical Committee for Animal Experiments at the University of Tokyo. Wild C57BL/6 mice were used in the GPC5 knockdown experiment. *Gt(ROSA)26Sortm9(CAG-tdTomato)Hze* mice were purchased from The Jackson Laboratory (Bal Harbor, ME). A loxP-flanked STOP cassette was used to prevent transcription of a CAG promoter-driven red fluorescent protein variant (tdTomato), all of which was inserted into the *Gt(ROSA)26Sor* locus. Mice specifically expressing Cre recombinase in podocytes under the regulation of a 2.5-kb fragment of the human NPHS2 promoter (Podocin-Cre) were generated by Holzman LB and colleagues^35^. F1 mice were obtained by breeding of *Gt(ROSA)26Sortm9(CAG-tdTomato)Hze* and Podocin-Cre mice. These mice shown to predominantly express the red fluorescent reporter protein in podocytes are named as podocin-tdTomato mice in this report. All the breeding process and gene expressions were confirmed step by step with PCR using specific primers.

### Preparation of TPFE–siRNA complexes

Tetra(piperazino)fullerene epoxide (TPFE) was synthesized by following the procedure reported previously (Supplemental Figure 5)^12^. As for siRNA, we used predesigned Gpc5 siRNA targeted against Gpc5 (NM_001107285.1_stealth_1056, Invitrogen), si*Shroom3* (sense strand, 5′-CAGAAGACCUCAGAAGAUAUCCGGA-3′ and antisense strand, 5′-UCCGGAUAUCUUCUGAGGUCUUCUG-3′) and si-Control (sense strand, 5′-CGGCAAGUCUCCCAAGGAGUU-3′ and antisense strand, 5′-UGAACUCCUUGGGAGACUUGC-3′). These siRNAs were purchased from Invitrogen. TPFE dissolved in 2 mM potassium chloride solution (pH 2.0) and siRNA dissolved in nuclease-free water were mixed to obtain a reagent-to-base pair ratio (R) of 10 or 15. The R value was calculated by dividing the nitrogen-to-phosphorus (N/P) ratio by 2. The mixture was incubated at room temperature for 5 min and then mixed with 10x PBS (pH 7.4; Gibco-Thermo Fisher, Waltham, MA) before injection.

### Analyses of urinary albumin and creatinine

We determined urinary and serum albumin levels with ELISA using a murine microalbuminuria ELISA kit (Albuwell M, Exocell), measured urine creatinine concentrations with L-type Wako Creatinine F (FUJIFILM Wako Pure Chemical Corp., Osaka, Japan). Albuminuria was determined as the ratio of urinary albumin to creatinine (g/gCr). All procedures were performed in accordance with the manufacturer’s protocols.

### Quantitative real-time RT-PCR

Total RNA was extracted using the TRIzol reagent. To obtain cDNA of the transcripts, the reverse transcriptase reaction was performed with 1 μg of the total RNA (High Capacity cDNA Reverse Transcription Kit, Applied Biosystems-Thermo Fisher). The quantitative real-time RT-PCR was performed with the synthesized cDNA and primers sets for *Shroom3* (forward, 5′-CCAGTTACCGGTCACAGCTT-3′; reverse, 5′-TTCCACATCCCCTCCCCTAG-3′); for β-actin as an internal control (forward, 5′-GGTCATCACTATTGGCAACGAG-3′; reverse, 5′-GTCAGCAATGCCTGGGTACA-3′) using the SYBR green system (SYBR Green I PCR Master Mix, Applied Biosystems-Thermo Fisher). The PCR products were analyzed using ViiA7 software (Applied Biosystems-Thermo Fisher).

### Immunofluorescence staining

Kidneys were flash frozen or paraffin-embedded with 10% formalin fixation; 10-µm frozen-sections were dried at room temperature for 10 min and fixed in 4% PFA at room temperature for 15 min. 3-µm paraffin-embedded sections were boiled in 10 mM sodium citrate buffer for 20 min for antigen retrieval after being deparaffinized in xylene and rehydrated gradient ethanol solution. Slides were blocked for 1 h in PBS with 1% goat serum. Before slides were incubated with anti-GPC5 antibody, additional blocking was performed with M.O.M. Blocking Reagent (MKB-2213, Vector Laboratories, Inc., Burlingame, CA) at room temperature for 30 min. Then the slides were incubated with primary antibodies at 4 °C overnight (guinea pig anti-nephrin [1:100] (Acris Antibodies, Herford, Germany, rabbit anti-*Shroom3* [1:400], or anti-GPC5 [1:50] (R&D Systems, Minneapolis, MN). After three washes in PBS, the slides were incubated with secondary antibodies for 1 h (goat anti–guinea pig Alexa 594 and goat anti–rabbit Alexa 488 or rabbit anti–mouse Alexa 488; all 1:500 dilutions) (Thermo-Fisher). The slides were washed and mounted using FluorSave Reagent (Calbiochem, La Jolla, CA). The sections were then examined visually using confocal microscopy (TCS SP5 II, Leica, Germany).

### Transmission electron microscopy

Small pieces of the renal cortex were fixed in 2.5% glutaraldehyde (EMS, Hatfield, PA) overnight, washed with 50 mM Tris-(hydroxymethyl) aminomethane-HCI buffer (pH 7.6), dehydrated in sucrose, fixed again in OsO_4_ and embedded into Epon. Ultrathin sections were examined under Hitachi electron microscope (H-1000).

### Cell culture

For in vitro knockdown experiments, we used rat glomerular epithelial cells transfected with either Stealth RNAi Negative Control Med GC (Invitrogen) or with predesigned *Shroom3* siRNA using Lipofectamine RNAiMAX (Invitrogen) according to manufacturer’s protocol. We incubated the cells for 48 h to allow knockdown of *Shroom3*. To determine the extent of knockdown, we quantified the protein level of *Shroom3* in transfected cells.

For immunofluorescence, cells seeded on collagen-coated culture slides were fixed with 4% paraformaldehyde (PFA, EMS), permeabilized with 0.2% Triton X-100 in PBS for 10 min and incubated for 1 h at room temperature with Alexa Fluor 488 Phalloidin (Invitrogen). After the slides were washed extensively with PBS, coverslips were mounted on slides with FluorSave Reagent (Calbiochem, La Jolla, CA).

### Quantification of lamellipodia

Whole-cell perimeters and perimeters with adjacent lamellipodia (an actin-rich fringe with fluorescence intensity gradually declining with the distance from the edge) of fixed, phalloidin-stained cells were traced using NIH Image J. Lamellipodia length and area were quantified as described^38^.

### Statistical analysis

Differences between the experimental groups were detected using Student’s *t* test or Wilcoxon test. Values are expressed as means ± SD; *P* < 0.05 was considered to indicate significance. Statistical analyses were conducted using R version 3.4.3 (R Development Core Team, Vienna, Austria). The analyses of GWAS were further detailed above.

## Supplementary information

Supplementary Information.
