# Supplementary material for: SHROOM3, the gene associated with chronic kidney disease, affects the podocyte structure
Source: Sci Rep. 2020 Dec 3;10:21103. doi: 10.1038/s41598-020-77952-9 (PMC7713385; doi:10.1038/s41598-020-77952-9)
Supplement: Supplementary file 1 — Supplementary Information. [file 41598_2020_77952_MOESM1_ESM.pdf]

SHROOM3, the gene associated with chronic kidney disease, affects the podocyte structure

Ryo Matsuura, M.D., Ph.D.[1], Atsuko Hiraishi, MSc[1, 2], Lawrence B. Holzman, M.D. [3], Hiroki Hanayama, MSc.[4], Koji Harano, Ph.D.[4], Eiichi Nakamura, Ph.D.[4], Yoshifumi Hamasaki, M.D., Ph.D[5], Kent Doi, M.D., Ph.D[6], Masaomi Nangaku, M.D., Ph.D.[1], *Eisei Noiri, M.D., Ph.D.[1,7]

Table of Contents

Supplemental Figure 1. Immunofluorescence staining of nephrin and GPC5 1
Supplemental Figure 2. The representative image of Podocin-tdTomato. 3
Supplemental Figure 3. The mRNA level of Shroom3 in different organs. 4
Supplemental Figure 4. An example of splicing site prediction for mutation at rs142647267 site. 5
Supplemental Figure 5. The structure of TPFE 6
Supplemental Table 1: Top 50 SNPs associated with eGFR. 7

Supplemental Figure 1. Immunofluorescence staining of nephrin and GPC5

Immunofluorescence staining of human kidney was visualized using confocal microscopy. GPC5 was detected as Alexa-488 fluorescence (green), and Nephrin was visualized using Alexa-633 fluorescence (red)

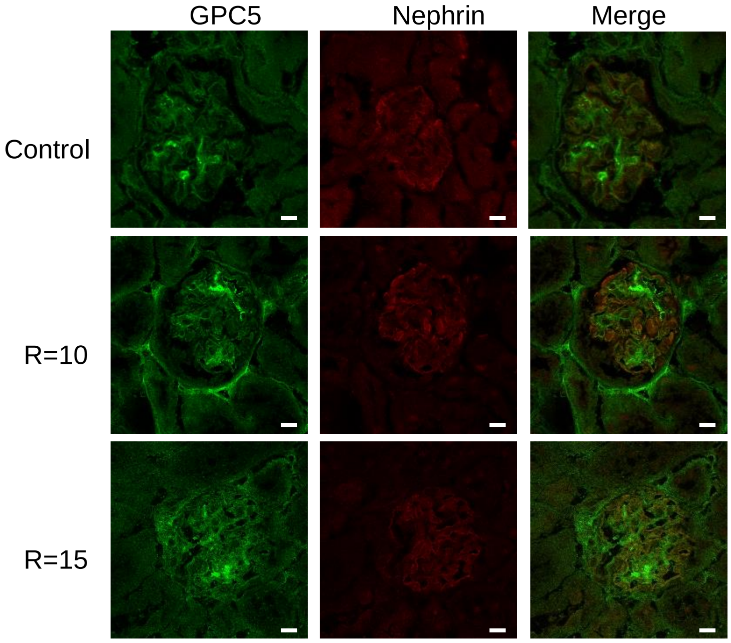

Supplemental Figure 2. The representative image of Podocin-tdTomato.

Shroom3 and Nephrin were also co-stained to confirm that tdTomato was expressed at podocyte. Bar = 25μm.

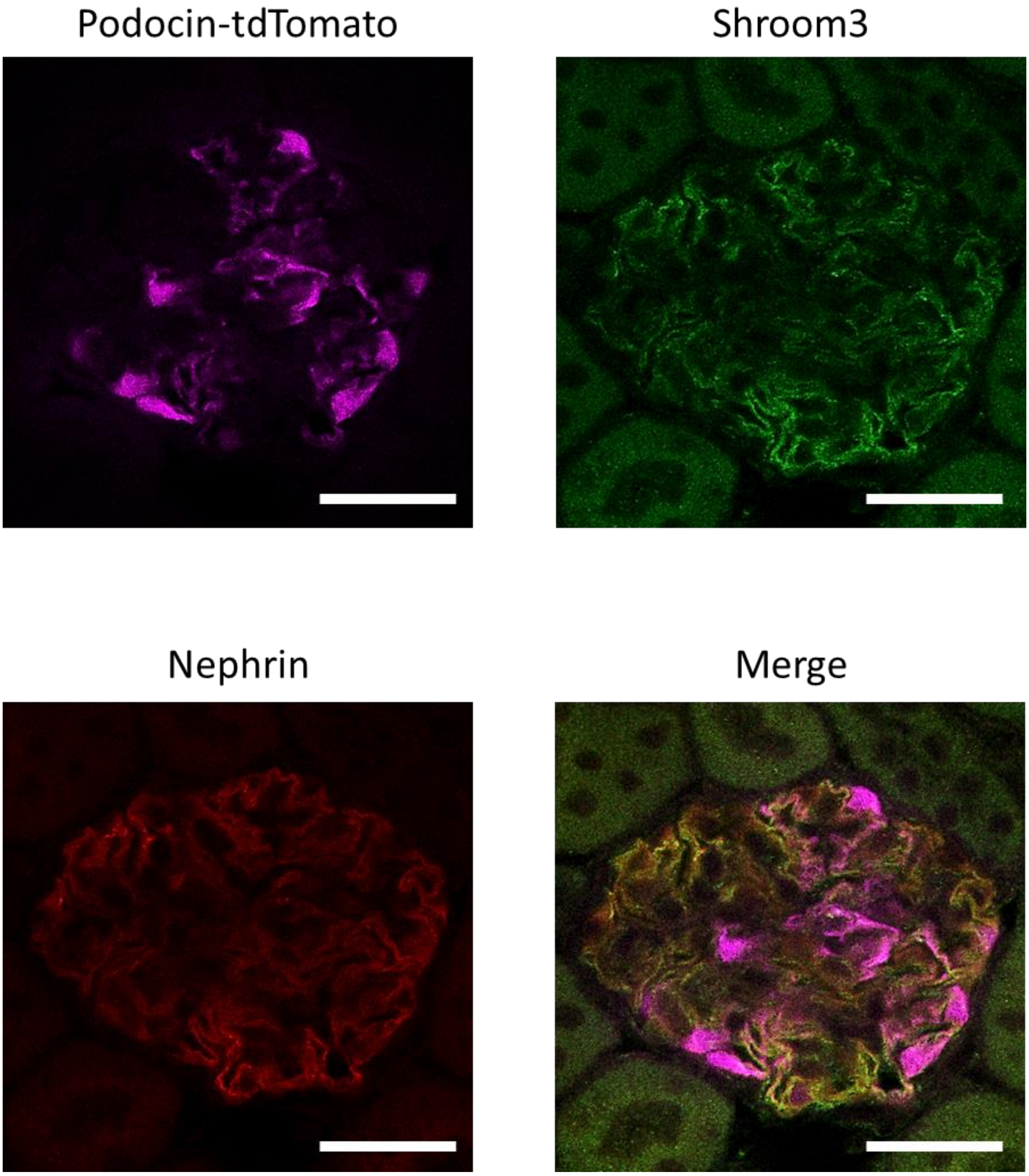

Supplemental Figure 3. The mRNA level of Shroom3 in different organs.

The RNA level in the kidney of mice with si-*Shroom3* was less, but not significantly, than that of mice with negative control siRNA. The RNA levels in other organs were not different. n=6-7 for each.

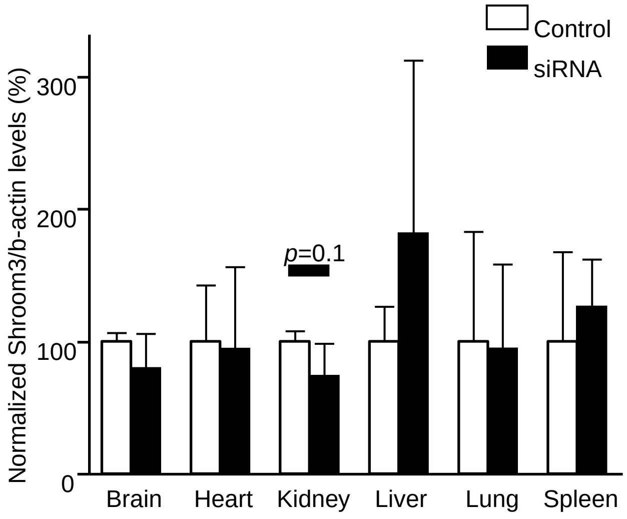

Supplemental Figure 4. An example of splicing site prediction for mutation at rs142647267 site.

This figure shows the predicted alternative splicing cite using a splicing site prediction tool called Alternative Splice Site Predictor (ASSP) (http://wangcomputing.com/assp/). The detailed method is described in the reference[1]. The upper column shows the normal sequence and the lower column shows the sequence with the rs142647267 variant. The red rectangle denotes the corresponding site. Compared to the normal sequence, the mutant variant possibly changes the splicing site and lowered the availability of the codon around this site. This prediction indicates that this variant would not generate Shroom3 properly.

Normal Sequence

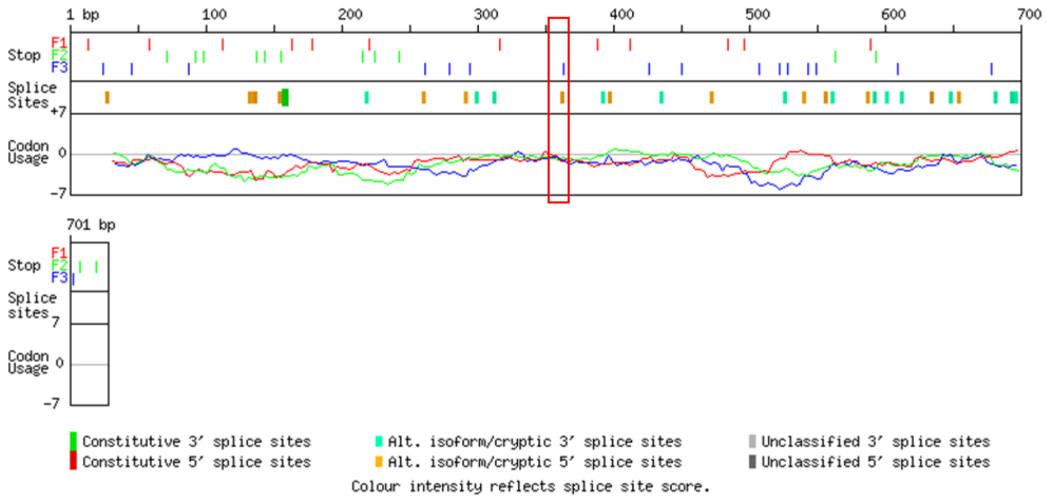

rs142647267 variant

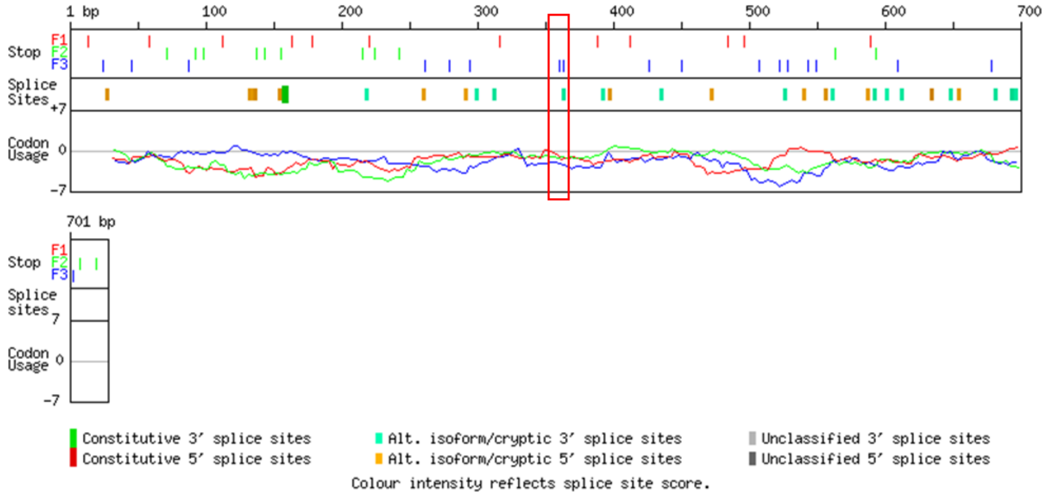

Supplemental Figure 5. The structure of TPFE

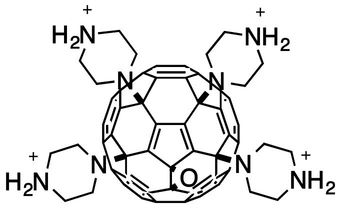

$4 \cdot CF_3CO_2^-$

TPFE

Supplemental Table 1: Top 50 SNPs associated with eGFR.

name	Chr	BP	A1	Freq1	MAF	Rsq	MainEffectTrueP	Gene
rs142647267	4	77401452	C	0.78334	0.21666	0.99907	3.33E-16	SHROOM3
rs10025351	4	77394095	C	0.78348	0.21652	0.99656	4.44E-16	SHROOM3
rs28394165	4	77394018	T	0.78349	0.21651	0.99631	4.44E-16	SHROOM3
rs4859682	4	77410318	C	0.78329	0.21671	0.99998	5.55E-16	SHROOM3
rs13146355	4	77412140	G	0.78309	0.21691	0.99679	5.55E-16	SHROOM3
rs10277115	7	1285195	A	0.68237	0.31763	0.95126	8.55E-15	UNCX
rs6950388	7	1270699	G	0.66752	0.33248	0.96273	9.53E-14	UNCX
rs10275044	7	1273845	A	0.6688	0.3312	0.99839	1.76E-13	UNCX
rs4724799	7	1274582	G	0.66882	0.33118	0.99891	1.77E-13	UNCX
rs6951209	7	1273821	T	0.66832	0.33168	0.99593	1.80E-13	UNCX
rs10032549	4	77398015	A	0.65743	0.34257	0.99995	3.91E-13	SHROOM3
rs4859681	4	77399164	C	0.65734	0.34266	0.9995	4.35E-13	SHROOM3
rs7654754	4	77409795	G	0.65709	0.34291	1	6.01E-13	SHROOM3
rs7656186	4	77409818	C	0.65711	0.34289	0.9999	6.37E-13	SHROOM3
rs7654978	4	77409945	G	0.65713	0.34287	0.99984	6.75E-13	SHROOM3
rs10029777	4	77411030	C	0.65743	0.34257	0.998	7.03E-13	SHROOM3
rs7675258	4	77413179	G	0.65716	0.34284	0.99494	7.09E-13	SHROOM3
rs7675217	4	77413142	G	0.65715	0.34285	0.99533	7.12E-13	SHROOM3
rs7674982	4	77412997	G	0.65714	0.34286	0.99611	7.13E-13	SHROOM3
rs7676094	4	77412894	C	0.65713	0.34287	0.9965	7.16E-13	SHROOM3
rs4318673	4	77412338	A	0.65712	0.34288	0.9969	7.20E-13	SHROOM3
rs13146163	4	77412091	G	0.6571	0.3429	0.99771	7.24E-13	SHROOM3
rs2137152	4	77411710	T	0.65733	0.34267	0.99731	7.24E-13	SHROOM3
rs2137153	4	77411660	G	0.6571	0.3429	0.99848	7.27E-13	SHROOM3
rs10029860	4	77411101	C	0.65712	0.34288	0.99904	7.29E-13	SHROOM3
rs61943010	12	111354623	C	0.78836	0.21164	0.99071	8.39E-13	MYL2
rs61943000	12	111354106	T	0.78841	0.21159	0.992	8.46E-13	MYL2
rs61942999	12	111353762	C	0.78869	0.21131	0.99907	8.88E-13	MYL2
rs2301610	12	111353556	A	0.78872	0.21128	0.99972	8.90E-13	MYL2

rs2078847	12	111352478	A	0.78868	0.21132	0.99948	8.91E-13	MYL2
rs11065770	12	111351937	C	0.78866	0.21134	0.99939	8.92E-13	MYL2
rs916164	12	111351599	A	0.78865	0.21135	0.99929	8.94E-13	MYL2
rs11065769	12	111351439	T	0.78863	0.21137	0.9992	8.95E-13	MYL2
rs2071629	12	111351186	C	0.78862	0.21138	0.99911	8.97E-13	MYL2
rs2137154	4	77411501	C	0.65507	0.34493	0.99276	1.19E-12	SHROOM3
rs11722924	4	77396854	G	0.66428	0.33572	0.97508	1.37E-12	SHROOM3
rs3825389	12	111350771	C	0.78769	0.21231	0.99972	1.72E-12	MYL2
rs3782889	12	111350655	A	0.78769	0.21231	0.99982	1.73E-12	MYL2
rs3782890	12	111350531	G	0.78766	0.21234	0.99926	1.73E-12	MYL2
rs3782891	12	111350457	G	0.78763	0.21237	0.9987	1.73E-12	MYL2
rs11065766	12	111349849	A	0.7876	0.2124	0.99814	1.73E-12	MYL2
rs12231049	12	111349223	A	0.78757	0.21243	0.99759	1.74E-12	MYL2
rs35449439	16	20385182	G	0.78045	0.21955	0.99356	1.15E-11	PDLT,UMOD
rs4408552	16	20383531	A	0.78047	0.21953	0.99152	1.16E-11	PDLT,UMOD
rs34532024	16	20385148	C	0.78046	0.21954	0.99308	1.16E-11	PDLT,UMOD
rs34495073	16	20385144	T	0.78046	0.21954	0.9926	1.16E-11	PDLT,UMOD
rs35208507	16	20388929	A	0.78037	0.21963	0.99352	1.21E-11	PDLT,UMOD
rs62034970	16	20383049	G	0.78105	0.21895	0.96578	1.41E-11	PDLT,UMOD
